# Validation of remote assessment of preschool children's anthropometrics and motor skills

**DOI:** 10.3389/fdgth.2023.1168618

**Published:** 2023-07-13

**Authors:** Alyssa M. Button, E. Kipling Webster, Chelsea L. Kracht, Chelsea Hendrick, Anthony Okely, Kar Hau Chong, Penny Cross, Amanda E. Staiano

**Affiliations:** ^1^ Division of Population and Public Health Science, Pennington Biomedical Research Center, Baton Rouge, LA, United States; ^2^ Department of Kinesiology, Recreation, and Sport Studies, The University of Tennessee, Knoxville, TN, United States; ^3^ Early Start and School of Health and Society, Faculty of the Arts, Social Sciences and Humanities, University of Wollongong, Wollongong, NSW, Australia

**Keywords:** assessment, remote, motor skills, anthropometrics, telehealth, telemedicine

## Abstract

**Introduction:**

Remotely delivered treatment and research procedures were rapidly adopted in response to the COVID-19 pandemic. However, it is unclear if these measures are valid. The purpose of this study was to compare the validity of anthropometry and motor skill proficiency measurements collected in a remote-setting to in-person setting among a sample of children ages 3–4 years.

**Methods:**

Child anthropometry and motor skill performance were measured in-person by trained assessors and by parents at home with remote supervision via videoconference by trained assessors. The following measures from the National Institutes of Health Toolbox were collected: anthropometry (height and weight), manual dexterity/manipulation (9-hole pegboard), motor coordination and agility (supine timed up and go), lower body strength (standing long jump), and postural stability (one-leg standing balance). Differences in expert and parent-based measurements were assessed using Bland-Altman plots, paired samples t-tests, and Pearson correlations.

**Results:**

A total of *n *= 14 children completed the assessments. No significant differences were observed between measurement locations for weight and motor skills (*p *> .05). Remote measurement of height (M = 101.1 cm, SD = 5.40) was significantly greater than in-person measurements (M = 98.2 cm, SD = 5.16); *p *< .0001.

**Discussion:**

Remote measurements of motor skills and weight are valid assessments for researchers and clinicians to utilize in young children. Remote assessment with guidance offers comparable and valid estimates as in-person assessment, potentially offering a solution to resource-constricted barriers in research and access to care. There is an opportunity for researchers to fine-tune remote height and individual-level assessment strategies.

## Introduction

Telehealth is the delivery of health care, health education, and health information services via remote technologies (1). In response to the COVID-19 global pandemic, health care providers were required to rapidly adopt, modify treatment for, and utilize a telehealth system of providing care (2). This was done to comply with social distancing recommendations for disease prevention (3). Telehealth and remote services also help provide services to rural and historically underserved communities, including Black, Hispanic, and Latino communities who have been disproportionately affected by COVID-19 (4, 5). Additionally, providers may use these services to close the gap between pediatric developmental-behavioral service demands and the lack of available providers (6).

While telehealth describes remote treatment, remote delivery refers to the modality of treatment- i.e., providing interaction (not required to be treatment-related) which can be accessed from two separate locations. Evidence shows the reliability and validity of remote services in providing effective interventions in the areas of occupational therapy (7) and motor skill development among children (8). Early development and intervention for fine and gross motor skills are critical given their positive associations with physical activity (9, 10), academic achievement (11, 12), and daily living skills (13). Further, pediatric obesity is associated with motor skill declines (14), indicating motor skills as an important target of pediatric weight management intervention (15). In the midst of the COVID-19 pandemic, preschool children, particularly those with obesity, experienced the greatest increases in body mass index (BMI) compared to adolescents (16). There is now a call for ample opportunities for sufficient physical activity and nutrition among this age group (16). Remote technologies may be a valuable tool to address this need.

Remote treatment procedures in pediatric populations are increasingly recognized as feasible and effective (17), but there is a major gap in the literature of evidence-based measurement and assessment procedures conducted remotely (17, 18). Remote motor skills interventions in pediatric populations are supported by evidence (8), and telemonitoring of motor skills has shown promising preliminary feasibility results (18, 19). To our knowledge, no studies are available assessing the validity of measurements for motor skills conducted remotely compared to in-person assessments. Similarly, anthropometric assessment modality comparisons are currently underreported, but preliminary data demonstrate promising results using portable stadiometers (20). There is a need for validated remote assessment of anthropometric and motor skills, particularly for researchers examining pediatric development and health outcomes. The purpose of the current study was to examine the validity of remotely supervised, parent-collected, at-home measurement of children's motor skills and anthropometry compared to the gold standard of in-person assessment by a trained assessor. Given the effectiveness of remote services in treatment administration, and the preliminary evidence for measurement feasibility, we hypothesized no significant differences in motor skills and anthropometric measurements between in-person and remote conditions.

## Methods

### Participants

The validation study was embedded within the “Indoor Active” pilot study and provided data for the SUNRISE International Surveillance Study. SUNRISE is an international cross-sectional study examining 24-h movement behaviors and developmental/health outcomes in the early years of life across geographically, culturally, and economically diverse countries (21). Pre-school children, ages 3–4, were recruited for this pilot study. Parents were recruited from a metropolitan area in the Southeastern United States via email listserv, social media, and community contacts for their child to participate. Indoor activity toys and up to $25 was offered to those children who completed the study. To be eligible for the study, children needed to be between the ages of 3–4 years old and the family needed to have access to a Wi-Fi-enabled streaming device (e.g., cellphone, tablet, computer and web camera). Children with parent-reported mobility limitations were excluded.

### Procedures

This validation study utilized a within-subjects design. Three visits were completed as part of the study “Indoor Active” study procedures (IRB # 2021-017-PBRC). The first visit was in-person at a laboratory setting, where informed parent consent and verbal child assent were obtained. At this visit, parents completed a demographics survey and child anthropometrics and motor skills measurements were collected by a trained assessor. At the end of this first visit, parents were provided with equipment for the remote measurement, with the same instructions provided in both written and video formats for completing the measurements ahead of the scheduled visit. Parents were provided a tripod (in case of using phone for remote visit), calibrated scale, a carpenter square and tape measure for height measurements, painter's tape, and a pegboard with 9 pegs. Written and video recorded instructions for collecting height and weight information were modified from the World Health Organization (WHO) guidelines to distribute to parents (22). Parent instructions for administering motor skills measurements were modified from the National Institutes of Health (NIH)- Early Childhood Battery (23). A video was created by trained assessors to provide examples for parents of how to accurately demonstrate the skills their child.

The second visit was a remote session conducted via secure video conferencing and was recorded. This visit occurred approximately one week from the first visit. The parents used their own Wi-Fi enabled device (e.g., cell phone, laptop). A trained assessor guided parents over video conference through each measurement (anthropometrics). Child anthropometrics were measured by the parents. The trained assessor remotely viewed administration and entered parent-reported data in real-time. The third/final visit took place remotely approximately one week from the second visit. During this visit, a trained assessor guided parents over video conference through each motor skills measurement and managed the stopwatch for timed skills. Child motor skill performance was measured by the parents, with the trained assessor remotely viewing and entering parent-reported data in real-time. All procedures followed the SUNRISE protocol (21).

### Measures

#### Anthropometry

At the first visit, a trained assessor collected child weight to the nearest 0.1 kg using an electronic, calibrated scale (Etekcity, China) and collected child height to the nearest 0.1 cm using a portable stadiometer and in accordance with World Health Organization guidelines (22). To measure weight, children were instructed to remove heavy layers of clothing as appropriate, to remove socks and shoes, and to stand still in the middle of the scale until the weight appeared on the display. To measure height, children were instructed to stand on the baseboard with feet slightly apart, with the back of their head and body touching the vertical stadiometer and their trunk aligned above their waist.

At the second visit (remote), an assessor remotely observed and recorded parents' report of weight measurement to the nearest 0.1 kg using a study provided electronic, calibrated scale. The assessor remotely observed and recorded parents’ report of the child's height to the nearest 0.1 cm using the provided materials (i.e., tape measure, carpenter square).

#### Motor skills

SUNRISE motor skill assessments were selected from the NIH Toolbox (23). This battery of assessments is included as a key domain in the NIH Toolbox for Assessment of Neurological and Behavioral Function. The subdomains measure dexterity, strength, balance, locomotion, and endurance. This battery has demonstrated excellent test-retest reliability with all measures meeting ICC > 0.80, and demonstrating criterion validity of *r *> 0.75 (23). To measure lower body strength at the second remote visit (third visit), children were observed by parents to perform a *standing long jump.* Parents were instructed to have the children stand with their toes behind a taped line marked on the floor, and then to jump with two feet together as far as they can, and to land on two feet. For the in-person visit (visit one), an assessor provided the same instructions as the parent gave during the remote visit. This task consisted of one practice and two test trials. Distance from the child's heel of the foot that was closest to the line and the front of the line was measured, and the child's greatest distance in centimeters between the two test trials were recorded for analyses.

A *supine-timed up and go (S-TUG)* was performed to assess motor coordination and agility. For the remote visit, parents were instructed to mark a line 3 meters across from any solid structure with tape or chalk, where they then attached a sheet of paper with a large target on it to the wall/structure at the child's eye level. Similarly, during the in-person assessment, a line was marked three meters from a wall with a target marked on the wall at the child's eye level. The child was instructed to lie supine (on their back) with the heels of their feet on the line. When instructed “go,” the child was required to get up as quickly as possible, run and touch the target, and then run back across the 3 m line as quickly as possible. Timing started when the assessor said “GO” and stopped as soon as the child's torso crossed the taped line. This task consisted of one practice and two test trials, with the child's fastest in seconds of the two test trials used for analyses.

Children completed a *one-legged standing balance test* to measure postural stability. Children were instructed to stand on one leg for up to 30 s. Children were required to keep the standing leg fixed but were allowed to keep the free leg in any position if it was off the floor and not hooked around the standing leg. Timing was started when the free leg left the floor and was stopped if the child moved the standing leg, hooked the free leg around the other leg, touched the free leg or supporting surface with their hands, or if the 30 s was complete. The test was then completed on the other leg. This task consisted of one practice trial and two test trials (per leg), and the length of time that the child balanced on each leg during the test trials were recorded and averaged for analyses.

Children completed the *9-hole pegboard test* (PAT-A8515, Sammons Preston, Illinois, USA) to measure manual dexterity and manipulation. During this test, children were timed as they picked up nine pegs one at a time and inserted them into the pegboard (31.1 cm × 26.0 cm × 4.3 cm) and then removed them using their right hand. The test was then repeated with their left hand. The timer began as soon as the assessor said “GO” and was stopped as soon as the final peg was placed back in the pegboard well. This task consisted of one practice trial and one test trial (per hand). The time in seconds for each hand was recorded and used for analysis.

#### Demographics

Parents responded to questions about sociodemographics based on a modified version of the WHO STEPS Survey (24).

### Statistical analyses

Prior to analyses, data were checked for normality. Means and standard deviations were calculated for both remote and in-person measurements. Bland-Altman plots were used to measure the absolute agreement between remote and in-person measurements. In addition to the calculation of error (remote—in person) and absolute error (|remote—in person|), paired samples *t*-tests were used to determine if there were differences between remote and in-person measurements. Cohen's *d* (25) effect sizes were calculated to determine the meaningfulness of the significant differences. Pearson correlations were used to analyze the degree of similarity between in person and remote measurements (25). All analyses were completed using SAS 9.4 software (26).

## Results

Fourteen children completed the anthropometric measurements and 13 completed the motor skills measures, with one child refusing to complete the motor skills measures at both the remote and in-person timepoints. Most participating children were male (57%), and average age was 3.4 ± 0.5 years. Participant characteristics are presented in Table 1.

**Table 1:** Descriptive characteristics.

	Mean (SD)	*n* (%)
Child	*N* = 14	
Age (years)	3.4 (0.5)	
Sex		
Male		8 (57)
Female		6 (43)
Parent	*N* = 14	
Age (years)	34.9 (3.7)	
Sex		
Male		1 (7)
Female		11 (79)
Did not respond		2 (14)
Racial Background		
European		11 (79)
Multiracial		1 (7)
Did not respond		2 (14)
Educational Attainment		
Secondary or high school		1 (7)
Vocational education		4 (29)
Tertiary education		8 (57)
Did not respond		1 (7)

Means and standard deviations for differences between measurement location are presented in Table 2.

**Table 2:** Comparison of Measurements by location.

Measurement	In-Person	Remote	Difference	*p*
	M(SD)	M(SD)		
Height	98.2 (5.2)	101.1 (5.4)	2.9 ± 1.1	<.0001*
Weight	16.6 (2.5)	16.6 (2.7)	0.02 ± 0.4	0.88
9-hole pegboard (right)	38.9 (12.3)	36.7 (15.0)	0.8 ± 21.5	0.90
9-hole pegboard (left)	31.9 (20.0)	30.0 (18.3)	1.9 ± 28.1	0.81
Supine timed up and go	6.3 (1.1)	6.6 (1.2)	0.3 ± 1.2	0.37
Standing long jump	51.7 (21.7)	48.4 (20.0)	−3.4 ± 18.2	0.52
One-legged standing balance (right)	5.9 (7.7)	4.2 (2.2)	−1.8 ± 7.2	0.39
One-legged standing balance (left)	5.9 (3.2)	4.9 (5.45)	−1.0 ± 3.8	0.36

* Cohen's d = 0.5.

**Figure 1:**
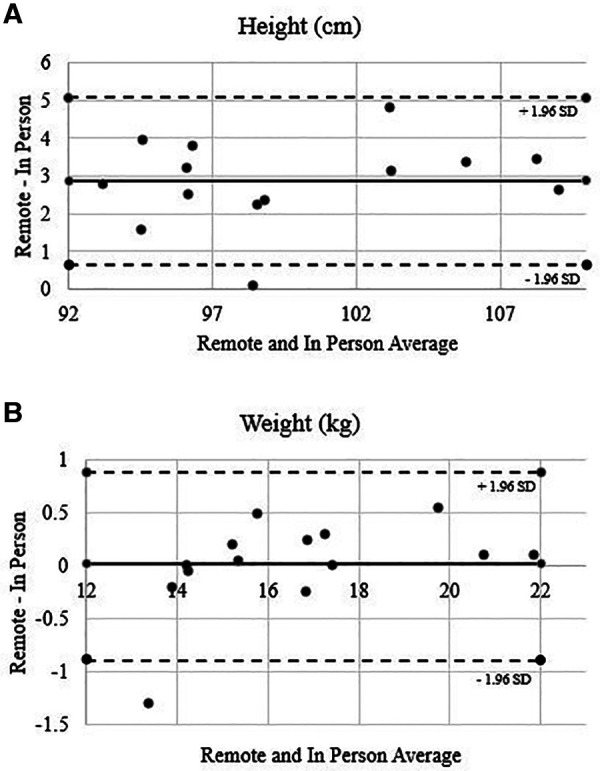
Height (**A**) and weight (**B**) Bland-Altman plots.

There was a significant difference in measurement of height by location, where the remote measurements were greater than the in-person measurements. This was a medium effect. The absolute error value was 2.22 cm, one participant had an error beyond the 95% limits of agreement (Figure 1A). Pearson correlations revealed large effects between in person and remote measurements (*r *= .978).

No significant differences were observed between remote and in-person measurements for weight with absolute error value of 0.87 kg. One participant error was beyond the 95% limits of agreement (Figure 1B). Pearson correlations revealed large effects between in person and remote measurements (*r* = .989).

**Figure 2:**
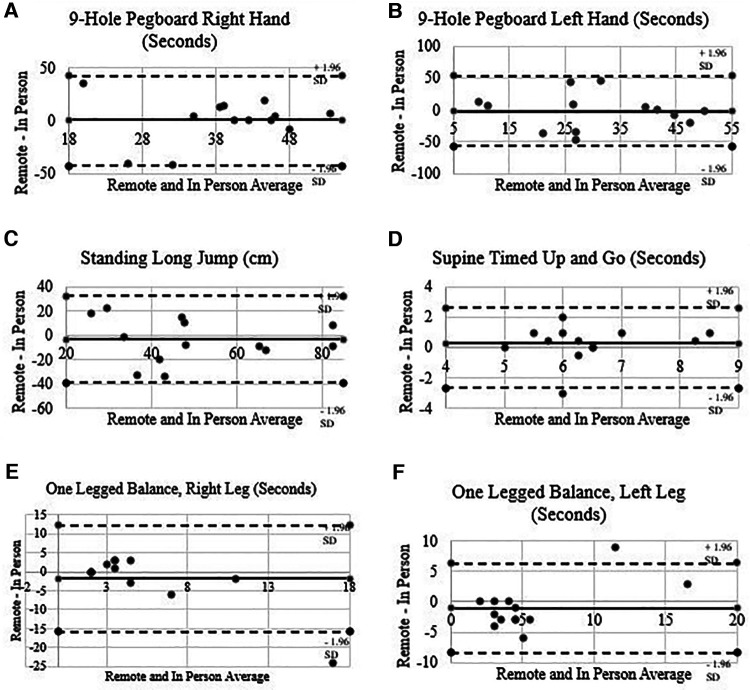
Motor skills Bland-Altman plots for 9-hole pegboard (**A**, **B**), standing long jump (**C**), supine timed up and go (**D**), and one-legged balance (**E**, **F**).

For the motor skills measurements, no meaningful differences were present between remote and in person-measurements for 9-hole pegboard (right and left-hand trials) with absolute error values of 42.10 and 55.10 s respectively. Pearson correlations revealed small effects between in person and remote measurement between 9-hole pegboard right hand (*r *= −.151) and 9-hold pegboard left hand (*r *= −.075). No significant differences were observed for measures of standing long jump with an absolute error value of 35.73 cm. Pearson correlations revealed large effects (*r *= .619). For each of these measures, no participants had errors beyond the 95% limits of agreement (Figures 2A–C, respectively). There was no significant difference in remote and in-person measurements of S-TUG with an absolute error value of 2.62 s and a medium effect size (*r* = .485). There were also no significant differences in remote and in-person measurements of one-legged (right and left-leg) standing balance with absolute error values of 14.05 and 7.38 s, and medium to large effect sizes (*r *= .375; *r *= .740) respectively. For each of these measurements, one participant had errors beyond the 95% limits of agreement (Figures 2C–E, respectively).

## Discussion

We compared two locations of measuring child anthropometrics and motor skills, one in-person by a trained assessor, and one remotely outside of the laboratory setting by the parent with a live assessor observing on video conference. When comparing group means, child weight and motor skills measurements did not differ by modality. Child height was significantly different by modality, as parents measured their child taller during the remote visit, than by a trained assessor in a laboratory setting. When this data was examined at the individual-level, remote measurements were less accurate compared with the standardized in-person measurements due to the distribution of data points. This study contributes to the dearth of knowledge on the validity of remote assessment procedures for measuring child anthropometrics and motor skills. Overall, our results suggest that child procedures typically used for in-person measurement of group comparisons can be amenable to remote administration at home.

One result of this study demonstrated discrepancy between in-person and remote height measurements. Reliable height measurements have presented a challenge within pediatric telemedicine research (27, 28), as height for research studies and clinical purposes are typically measured by trained assessors in-person (29). Advances in automated height measurements via cellphones or tablet-based techniques may be incorporated to decrease errors in measurement (28). Ghosh-Dastidar and colleagues (2020) also demonstrated reliable height measurements using portable stadiometers (20). It is possible that the accuracy of these stadiometer tools are more robust compared to protocols using carpenter's square and measuring tape. We encourage more standardized equipment to be used in future studies when possible/feasible to distribute to homes and/or other remote locations. Another consideration, depending on treatment or research target, is a combination approach of utilizing both remote measurements for the measures that are more reliable, and in-person for those that are less reliable remotely (i.e., height). There is a need and an opportunity for researchers and clinicians to fine-tune remote height assessment procedures and supporting parents to collect accurate heights for the purposes of remote measurement.

The present study did not find significant differences between weight and motor skills measured in-person compared to remote. These results are promising for the utility of remote measurement, especially given the impact that fundamental motor skills have on future movement behaviors (30), health behaviors (31–33), physical activity (34), and weight status (35). Valid weight measurement is critical as there is an increasing demand for remotely delivered weight management interventions and weight is an important marker of children's health (29, 36). Validation of remote measurement procedures are needed urgently to support the rapid adoption of telehealth delivery (17). Moreover, other researchers are finding remote measurement instruction to be feasible and acceptable to potential assessors (37). Results from this study suggest that these measurements are valid primarily for use at the group-level. More work is needed in validating procedures appropriate for individual-level measurement.

This study leveraged an international surveillance effort and the unique opportunity of the remote assessments during the COVID-19 pandemic to test common child measurements. We were able to demonstrate validity of weight and motor skills remote measurements among preschool samples, an important contribution to pediatric weight management and motor skills intervention/treatment. We also recognize several limitations. First, there is potential of order effects. For motor skills, the procedures consistently started with remote measurement first and then in-person measurements second, whereas height and weight were collected in-person first and then remotely. Parents were not provided their child's height or weight at the in-person visit to minimize bias. Order effects may be especially evident for the one-legged balance where children were observed to balance longer at the in-person session during the third visit, though for the pegboard task they took longer to complete at this same visit. It would have been optimal to have randomized the order, but this was not feasible within the overall study protocol. Second, the cost of providing materials to families for remote measurements may limit the ability to reach larger samples. Supplies, including shipping, are costly and there is potential for losing or damaging supplies in transit. Third, there were not enough measurements obtained to calculate relative reliability. However, we did use standardized in-person measures as a comparison of the remote measurement validity. Fourth, generally, this was a predominately white and educated population, who may have more resources (e.g., time, connectivity) than others to administer these measurements. The COVID-19 pandemic disproportionately affected people without access to the internet (38, 39). Long-term solutions are required to allow everyone the opportunity to administer remote measurements to relieve burden of travel and other circumstances. Access to internet connectivity and technological literacy all remain barriers to the use of remote assessment and telehealth that must be considered when designing treatment and assessment tools (40). Clinicians and researchers may consider partnering with community organizations and healthcare providers to overcome these barriers.

Anecdotally, our team experienced challenges with parent visit preparation and technical difficulties during remote visits. To address these challenges, we offer the following recommendations: (1) give parents plenty of time to manage children's and siblings' behavior when attention spans are limited, or disruptions occur; (2) live video visits during remote assessments are critical for staff to assist parents virtually; (3) parents benefit from brief written and video instructions prior to visit including to prepare the space at home; and (4) some parents require a tripod to position their smartphone camera for stable viewing by the virtually connected trained assessor. Another consideration is the variation of settings and environments in remote measurement. Households with limited space or clutter may present challenges with the standing long jump task, for example, and there is a chance that carpeting, or baseboards may interfere with height and weight measurement, as compared to more “ideal” conditions and standardization that a research lab or clinical setting affords. Future research may benefit from understanding how different demographics, physical household characteristics, and parental characteristics may alter the validity of data collected at home via remote procedures (41). Remote measurements were observed and guided by trained assessors; therefore, we cannot speak to the validity of these procedures when conducted *independently* by parents and caregivers. Overall, results from this study suggest that remote measurement of child motor skills and weight is valid compared to in-person assessment in a research laboratory. Remote measurement options may help to reach more representative populations for both clinical and research purposes (e.g., children who do not attend childcare, rural families, children from other geographical regions) and overcome barriers to accessing in-person assessments.

## Data Availability

The raw data supporting the conclusions of this article will be made available by the authors, without undue reservation.
